# Disparities in multi-modal spatial access to primary and specialty care in U.S. neighborhoods: A cross-sectional and temporal analysis of health services

**DOI:** 10.1371/journal.pone.0330427

**Published:** 2025-09-17

**Authors:** R. Blake Buchalter, Paul R. Gunsalus, Madeleine M. Blazel, Michael W. Kenyhercz, Jarrod E. Dalton

**Affiliations:** 1 Department of Epidemiology, University of Alabama at Birmingham School of Public Health, Birmingham, Alabama, United States of America; 2 Genomic Medicine Institute, Cleveland Clinic, Cleveland, Ohio, United States of America; 3 Cleveland Clinic Lerner College of Medicine, Case Western Reserve University, Cleveland, Ohio, United States of America; Allegheny General Hospital, UNITED STATES OF AMERICA

## Abstract

Spatial access to care describes the ability of populations to travel to healthcare providers. Existing spatial access to care studies frequently utilize metrics constructed using car-specific travel to providers, potentially underestimating access in populations using other travel modalities (i.e., walking/transit). We present a novel multi-travel modality spatial access to care composite measure, a corresponding temporally representative open-source data resource, and a stratified analysis of neighborhood rural/urban and socioeconomic disparities. We utilized quarterly 2016–2020 Centers for Medicare and Medicaid Services Doctors and Clinicians National Downloadable Files to compute temporally representative multi-modal spatial accessibility to 56 healthcare provider classifications in U.S. census tracts and block groups. We performed tract-level analyses stratified by 2010 Rural-Urban Commuting Area Codes and 2016 Area Deprivation Index for a subset of provider types. We found that 1) non-metropolitan areas have poorer multi-modal spatial access to primary (P < 0.001), specialty (P < 0.001), pulmonology (P < 0.001), nephrology (P < 0.001), and cancer care (P < 0.001) than metropolitan areas both cross-sectionally and over time and 2) multi-modal primary care access was substantially poorer than multi-modal specialty care access in 2016 and this trend did not improve over time. We also produced a data resource for many provider types and an interactive spatial access map for a subset of provider classifications. After stratifying, we found significant disparities in spatial access to primary care, specialty care, pulmonology, nephrology, and cancer care in non-metropolitan areas. Our multi-modal spatial access measure and corresponding data resource may be useful for population health and clinical studies, public health entities, community stakeholders, health systems, and policymakers.

## Introduction

Access to healthcare is a multifaceted phenomenon that includes dimensions of affordability, availability, accessibility, accommodation, and acceptability [1]. Many of these aspects are difficult to quantify at the population level. For example, accommodation relates to the ability of a provider’s office or hospital to meet the individual patients’ preferences and constraints. In contrast, geographic accessibility, or how easily patients can physically travel to their physicians, is more straightforward to measure. Geographic accessibility to healthcare, also known as spatial access to care, has been studied for over twenty years [2]. Yet, these studies have been primarily for primary care providers and other limited provider types [3,4]. The floating catchment area (FCA) method and derivatives are commonly employed to study spatial access to care, which utilize a distance or travel time matrix, often by car, to create weighted provider-to-population (P2P) ratios for small areas [2,5–9]. We introduce an evolution in the form of a composite measure and corresponding open-source dataset, which weights spatial access by how populations in small areas actually travel (e.g., by transit, walking, or driving). Multi-modal spatial access to care metrics have been formulated previously in the literature, but most 1) are for areas outside of the U.S. or limited U.S. states or regions, 2) have computed metrics for only limited provider types, or 3) do not publicly release data for the metrics [10–21]. We specifically target these shortcomings and also compute spatial access to care metrics nationally for block groups (BGs) in the contiguous U.S.

U.S. rural, suburban, and urban populations utilize different travel modes in daily life and accessing healthcare [22,23]. Rural residents specifically have considerable difficulty in accessing alternative transportation modalities (e.g., transit) [24], yet according to the Bureau of Transportation Statistics, over 1.5 million U.S. rural residents utilize non-car travel modalities for daily commuting [25]. Even within urbanized areas, access to transit routes and walkability can vary widely [26], potentially resulting in differences in how populations travel to both primary care providers and specialty care providers. Existing studies also suggest that walkability and public transit access in socioeconomically under-resourced areas vary by U.S. region [27–29], which could influence access to care for lower income communities since rates of car ownership are lower [30,31]. Rural residents have difficulty accessing healthcare providers for both preventive health services and treatment [6,7,32,33], which may be contributing to widening disease burden and mortality among rural populations [34–37]. Yet, to our knowledge, no studies examining rural/urban strata have utilized multi-modal spatial access metrics to account for various modes of travel and for different types of healthcare providers. Therefore, quantifying multi-modal spatial access to care across rural-urban and socioeconomic continuums is an important step towards better understanding of where improving access could have the strongest benefit. Identifying neighborhoods with poor multi-modal access to primary and specialty care also would provide crucial information for lawmakers to target funding initiatives and programs, such as expansion of the Conrad 30 J-1 visa waiver program for foreign medical graduates, to improve access to providers.

Overarching objectives of our analyses were to create a neighborhood-level multi-modal spatial access to care metric, examine disparities by rurality and socioeconomic position, and produce a spatial access data resource useful for population health and clinical studies, public health entities, community stakeholders, health systems, and policymakers.

## Materials and methods

We used a comprehensive physician location dataset (estimated 98.9% physician coverage) [38] from the Centers for Medicare and Medicaid Services (CMS) to construct a new spatial accessibility measure for an expansive set of provider classifications in nationwide contiguous U.S. census tracts and BGs. We applied novel travel modality weighting to create the first open-source temporally representative spatial access to care dataset and performed analyses stratified by Rural-Urban Commuting Area (RUCA) codes and Area Deprivation Index (ADI) values. Our composite measure relies completely on publicly available data and utilizes a ‘realistic’ routing algorithm accounting for egress times (i.e., walking to/from transit stations) [39]. Finally, we constructed an interactive map of census tract-level spatial access to care measures for primary care and specialty care providers.

### Data sources

We obtained data from six sources to build our spatial access to care measures: 1) physician addresses from quarterly 2016–2020 Doctors and Clinicians National Downloadable Files (NDFs) from CMS [40], 2) tract and BG population counts from 2016–2020 U.S. Census Bureau American Community Survey (ACS) 5-year estimates, 3) 2010/2020 tract and BG polygons from the U.S. Census Bureau’s TIGER/Line shapefiles database, 4) national car and walking network datasets from OpenStreetMap (OSM), 5) national transit network datasets collated from an open-source catalog of U.S. transit routes [41], and 6) 2016–2020 ACS tract and BG population commuting patterns for driving, walking, and transit. For the routing component, OpenStreetMap (OSM) is one of the largest free global map databases, where information needed for routing (e.g., road networks, speed limits, stop signs/lights) is sourced from surveys, satellite imagery, and other geodata sources [42]. BusMaps is an open-source database of global transit General Transit Feed Specification (GTFS) files, which derives its database from city, town, and other local level GTFS files [41]. NDF provider specialties were used to classify active providers, which is updated regularly for providers who practiced and billed Medicare or Medicaid in the previous year [40]. Details of provider geocoding can be found in the Appendix. CMS NDFs were selected over other potential sources due to consistent updates on practicing physicians, relative completeness owing to the ubiquity of Medicare/Medicaid billing, and previous studies using this data in the physician spatial access literature [40,43,44].

### Travel matrices and spatial access measures

We computed “realistic” travel times given network datasets from OSM (car and walking) and the open-source transit network catalog (transit) [39]. Specifications used in this operation can be found in the Appendix. We applied the enhanced two-step FCA (E2SFCA) method, used widely in the literature [6,8,9,45], to calculate spatial access for categories of providers for 2016–2020 quarterly time periods. E2SFCA computes a travel time step-weighted P2P ratio with an incorporated distance decay function, which helps to capture the notion that patient populations are more likely to visit providers nearer to their neighborhood (methodology in the Appendix) [5]. We categorized providers into 56 groups based on medical licensing board classifications with input from clinical providers at Cleveland Clinic. (classification data at https://github.com/SpatialEpidemiology/SACData/tree/main/Physician%20Classifications).

### Weighting

We used 2016–2020 ACS 5-year tract or BG-level population estimates of those that commute to work via driving, walking, and transit as a proxy for how patient populations travel to providers. Population sizes by travel modality were then converted to percentages, where the denominator was the sum of the populations commuting via car, transit, and walking. We applied these commuting population percentages towards a proportional weighting scheme of car, transit, and walking P2P ratios to create composite car-transit-walking P2P ratios for each tract/BG and quarterly time period. For example, if a given area had population commuting patterns of 85% driving, 10% transit, and 5% walking, then the resulting car-transit-walking P2P ratio was constructed from 85% of the car P2P ratio, 10% of the transit P2P ratio, and 5% of the walking P2P ratio (see equation in the Appendix).

### Study area and stratified analyses

The study area encompasses tracts and BGs spanning the entire contiguous U.S. We created primary care, specialty care, pulmonology, nephrology, and cancer care access maps for tracts from different U.S. regions and differing patterns of car commuting, including Washington, D.C.; Cleveland, OH; Houston, TX; San Francisco, CA; and Augusta, ME (supplementary maps at https://github.com/SpatialEpidemiology/SACData/tree/main/Figures). We performed stratified analyses comparing spatial access between classifications of 2010 U.S. Department of Agriculture RUCA codes and 2016 ADI quartiles in addition to percent change in spatial access between December 2016 and December 2019 (end of year provider files). 2020 was not utilized for percent change due to changes in tract boundaries in the decennial 2020 Census. Quarterly spatial access to care datasets for 2016–2020 for all 56 provider classifications can be found at github.com/SpatialEpidemiology/SACData. Finally, we produced a tract-level interactive map with 2016 and 2019 spatial access measures for primary care and specialty care providers, found at https://spatialepidemiology.github.io/SACData.

## Results

There were 388,595 primary care, 626,825 specialty care, 23,448 pulmonology, 16,670 nephrology, and 34,713 cancer care providers included in our analysis of 2016 U.S. health care providers, where the total number of health care providers included in our analysis was 2,303,812 (excluding dentistry and providers with undefined specialty). These counts include individual provider duplicates, as all provider locations were included in the analysis (e.g., providers with separate hospital and clinic locations).

We tabulated composite P2P ratios for December 2016 in the contiguous U.S., which had medians of 105.8 primary care providers, 177.6 specialty care providers, 5.8 pulmonology providers, 4.2 nephrology providers, and 8.0 cancer care providers per 100,000 population (Table 1). Medians for car-specific P2P ratios for 2016 are shown in Table 1. Across all 5 physician classifications studied, stratified median composite and car-specific spatial access to care values were significantly lower in micropolitan, small town, and rural RUCA-classified tracts in comparison to metropolitan tracts. Median composite and car spatial access values were much more scattered across ADI quartiles without defined patterns. Between 2016 and 2019, median composite spatial access to primary care providers increased by 15.0% nationally, while composite spatial access to specialty care, pulmonology, nephrology, and cancer care increased by 50.0%, 13.2%, 16.4%, and 20.3%, respectively. Changes in medians were not consistent across RUCA strata, where micropolitan and rural strata had significantly lower percent increases in primary care composite spatial access in comparison to metropolitan tracts, while micropolitan, small town, and rural strata had significantly lower percent increases in pulmonology, nephrology, and cancer care in comparison to metropolitan tracts. Notably, for primary care, rural tracts experienced 0% median increase in composite spatial access to care, while pulmonology, nephrology, and cancer care, micropolitan, small town, and rural tracts had 0% median increase in composite spatial access to care between 2016 and 2019.

**Table 1:** Composite and car-specific spatial access to care summary statistics by rural-urban commuting area code and area deprivation index strata.

	Primary care		Specialty care		Pulmonology		Nephrology		Cancer care	
	Providers per 100k population (median)	Difference in median (p-value)	Providers per 100k population (median)	Difference in median (p-value)	Providers per 100k population (median)	Difference in median (p-value)	Providers per 100k population (median)	Difference in median (p-value)	Providers per 100k population (median)	Difference in median (p-value)
Composite access, 2016	105.8	--	177.6	--	5.8	--	4.2	--	8.0	--
Metropolitan	114.3	Ref	195.7	Ref	6.6	Ref	4.8	Ref	9.2	Ref
Micropolitan	72.6	**41.7** **(<0.001)**	111.8	**83.9 (<0.001)**	1.8	**5.2 (<0.001)**	1.2	**3.6 (<0.001)**	3.4	**5.8 (<0.001)**
Small town	64.9	**49.4 (<0.001)**	74.2	**121.5 (<0.001)**	0.0	**7.9 (<0.001)**	0.0	**6.4 (<0.001)**	0.0	**9.2 (<0.001)**
Rural	34.2	**80.1 (<0.001)**	31.6	**164.1 (<0.001)**	0.0	**7.9 (<0.001)**	0.0	**6.4 (<0.001)**	0.0	**9.2 (<0.001)**
ADI 0–25%ile	109.9	Ref	205.4	Ref	6.6	Ref	4.5	Ref	9.4	Ref
ADI 26–50%ile	99.7	**10.2** **(<0.001)**	166.5	**38.9 (<0.001)**	5.2	**2.4 (<0.001)**	3.7	**0.8 (<0.001)**	7.3	**2.1 (<0.001)**
ADI 51–75%ile	127.9	**−18.0 (<0.001)**	204.4	**1.0 (0.001)**	7.2	**−0.6 (<0.001)**	5.9	**−1.4 (<0.001)**	10.4	−1.0 (0.251)
ADI > 75%ile	130.7	**−20.8** **(0.002)**	218.6	−13.2 (0.570)	7.8	**−1.2 (0.020)**	6.3	**−1.8 (<0.001)**	10.5	−1.1 (0.700)
Car access, 2016	97.5	--	163.3	--	5.3	--	3.9	--	7.5	--
Metropolitan	105.2	Ref	179.5	Ref	6.1	Ref	4.5	Ref	8.6	Ref
Micropolitan	69.8	**35.4 (<0.001)**	107.5	**72.0 (<0.001)**	1.8	**4.7 (<0.001)**	1.2	**3.3 (<0.001)**	3.3	**5.3 (<0.001)**
Small town	62.4	**42.8 (<0.001)**	71.5	**108.0 (<0.001)**	0.0	**7.3 (<0.001)**	0.0	**4.5 (<0.001)**	0.0	**8.6 (<0.001)**
Rural	33.1	**72.1 (<0.001)**	30.3	**149.2 (<0.001)**	0.0	**7.3 (<0.001)**	0.0	**4.5 (<0.001)**	0.0	**8.6 (<0.001)**
ADI 0–25%ile	98.8	Ref	182.3	Ref	6.1	Ref	4.1	Ref	8.7	**Ref**
ADI 26–50%ile	93.2	**5.6 (<0.001)**	155.1	**27.2 (<0.001)**	4.9	**1.2 (<0.001)**	3.5	**0.6 (<0.001)**	6.9	**1.8 (<0.001)**
ADI 51–75%ile	114.0	**−15.2 (<0.001)**	181.9	**0.4 (<0.001)**	6.4	−0.3 (0.242)	5.3	**−1.2 (<0.001)**	9.3	−0.6 (0.073)
ADI > 75%ile	109.4	−10.6 (0.596)	170.4	**11.9 (0.024)**	6.7	−0.6 (0.694)	5.2	**−1.1 (0.024)**	8.4	0.3 (0.362)
2016-2019 composite ∆ (% change)	15.0%	--	50.0%	--	13.2%	--	16.4%	--	20.3%	--
Metropolitan	15.5%	Ref	49.0%	Ref	20.6%	Ref	25.7%	Ref	26.9%	Ref
Micropolitan	17.1%	**−1.6% (<0.001)**	59.6%	**−10.6% (<0.001)**	0.0%	**20.6% (<0.001)** ^**†**^	0.0%	**25.5% (0.001)** ^**†**^	0.0%	**26.9% (<0.001)** ^**†**^
Small town	12.8%	2.7% (0.32)	78.4%	**−29.4% (<0.001)**	0.0%	**20.6% (<0.001)** ^**†**^	0.0	**25.5% (<0.001)** ^**†**^	0.0%	**26.9% (<0.001)** ^**†**^
Rural	0%	**15.5% (<0.001)**	35.7%	**13.3% (<0.001)**	0.0%	**20.6% (<0.001)** ^**†**^	0.0	**25.5% (<0.001)** ^**†**^	0.0%	**26.9% (<0.001)** ^**†**^
ADI 0–25%ile	15.0%	Ref	41.2%	Ref	19.2%	Ref	26.3%	Ref	26.2%	Ref
ADI 26–50%ile	15.5%	**−0.5% (0.001)**	52.3%	**−11.1% (<0.001)**	11.3%	**7.9% (<0.001)** ^**†**^	12.9%	**13.4% (<0.001)** ^**†**^	18.2%	**8.0% (<0.001)** ^**†**^
ADI 51–75%ile	13.3%	**1.7% (0.001)**	47.1%	**−5.9 (<0.001)**	15.8%	**3.4% (<0.001)** ^**†**^	21.1%	5.2% (0.075)^†^	24.2%	**2.0% (<0.001)** ^**†**^
ADI > 75%ile	11.0%	4.0% (0.06)	37.5%	3.7% (0.514)	13.8%	5.4% (0.343)^†^	18.4%	7.9% (0.322)^**†**^	30.4%	−4.2% (0.593)^**†**^

Bold denotes p < 0.05 derived from Wilcoxon rank-sum test. † denotes p-values calculated via zero-inflated Wilcoxon rank-sum test due to excess zeroes.

For specialty care spatial access, micropolitan and small town RUCA strata had significantly larger median increases in composite spatial access in comparison to metropolitan-coded tracts between 2016 and 2019, while rural-coded tracts had significantly lower increases in composite access, suggesting a trend among health systems to increase specialty care providers outside of centralized urban locations.

**Fig 1:**
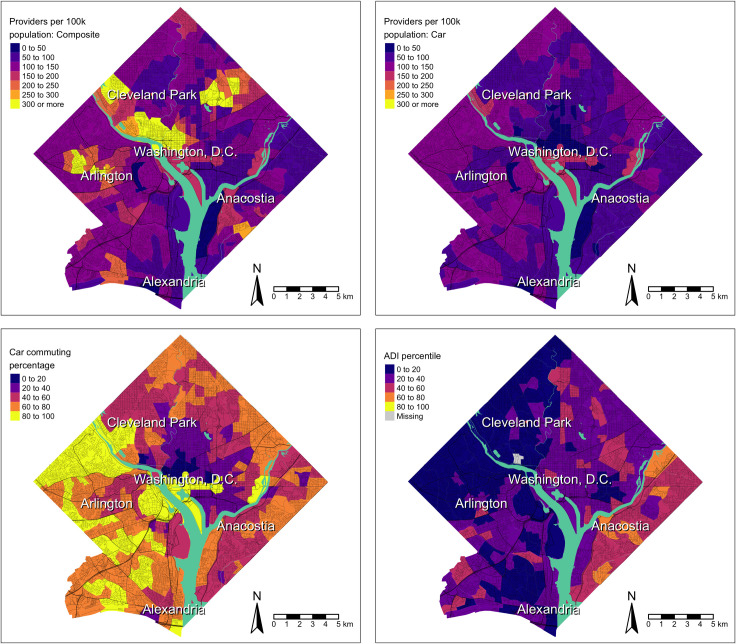
December 2016 travel modality-weighted car-transit-walking composite spatial access to primary care providers (A), December 2016 car-specific spatial access to primary care providers (B), 2016 car commuting percentage (C), and 2016 area deprivation index percentiles (D) in Washington D.C. metropolitan area census tracts.

**Fig 2:**
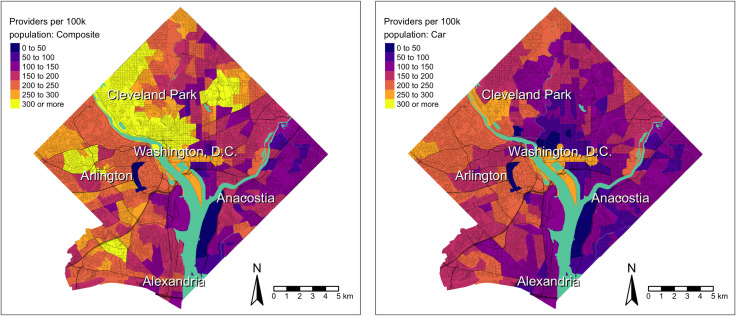
December 2016 Travel modality-weighted car-transit-walking composite spatial access to specialty care providers (A) and December 2016 car-specific spatial access to specialty care providers (B) in Washington D.C. metropolitan area census tracts.

As shown for the Washington, D.C. metro (Fig 1), car-specific spatial access to primary care providers (Panel B) likely underestimates access, as composite spatial access to primary care providers (Panel A), indicates substantially higher access where there are fewer people utilizing car travel (Panel C). There were no clear overlapping visual patterns when comparing primary care spatial access and area deprivation (ADI) in Washington, D.C. Both composite and car-specific access to specialty care providers were substantially higher than primary care provider access in 2016. In Washington D.C., composite and car access to specialty care providers followed similar geographic patterns to primary care access but was generally higher throughout the metro (Fig 2). Areas with the highest access (central D.C. and west of Arlington, VA) also had more sprawling access to specialty care providers than primary care providers. Given a considerable portion of the population in D.C. area utilizes transit or walking for commuting, access to care via car measure likely underestimated access in comparison to the composite measure.

**Fig 3:**
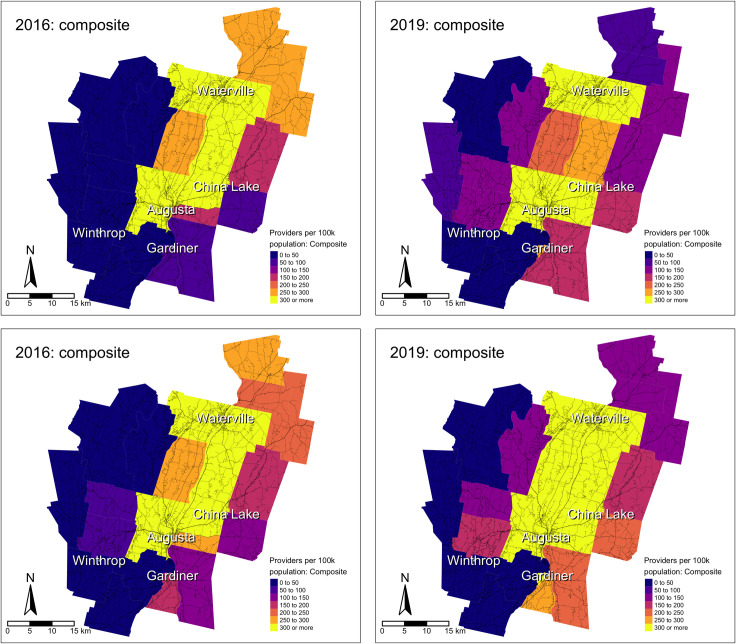
December 2016 travel modality-weighted car-transit-walking composite spatial access to primary care providers (A), December 2019 travel modality-weighted car-transit-walking composite spatial access to primary care providers (B), December 2016 travel modality-weighted car-transit-walking composite spatial access to specialty care providers (C), and December 2019 travel modality-weighted car-transit-walking composite spatial access to specialty care providers (D) in Kennebec County, ME census tracts.

The incongruities between access to specialty care and primary care continued to widen between 2016 and 2019, and these changes were particularly stark in non-metros. For example, in tracts coded as micropolitan, composite access to primary care providers increased by 17.1% from 2016 to 2019, while composite access to specialty care increased by 59.6% (Fig 3). Kennebec County, ME (Augusta, ME and surrounding areas) is a useful exemplar for these temporal differences, as the U.S. Office of Management and Budget and the U.S. Census Bureau classifies Kennebec County as a rural county [46], while individual tracts in the county are largely RUCA-coded as micropolitan, with one central tract in Augusta, ME coded as metropolitan. In Fig 3, primary care and specialty care composite access were roughly similar throughout Kennebec County in December 2016 (Panels A and C). In contrast, composite access to primary care providers increased slightly yet incongruently by 2019 (Panel B), while composite access to specialty care providers strongly increased, including in tracts further from the Augusta, ME urban core (Panel D).

## Discussion

We produced a novel multi-modal neighborhood-level spatial access to care measure and created the first temporally representative spatial access dataset for a variety of provider classifications in U.S. tracts and BGs for 2016–2020. Our analytical findings indicate non-metropolitan tracts had significantly poorer spatial access to primary care, specialty care, pulmonology, nephrology, and cancer care in 2016 and this trend worsened over time. We also found that that multi-modal spatial access to primary care providers was substantially lower than specialty care access in 2016 and worsened over time. We did not identify clear spatial access disparities across area deprivation values. Our findings and produced data resource are likely to be useful for clinical and population health studies, public health entities, community stakeholders, health systems, and policymakers in studying the effects of geographic accessibility to healthcare on health conditions/outcomes and targeting of community efforts, policy programs, and funding towards disparities in travel-related access. We expect to continually update this spatial access to care resource for subsequent time periods (e.g., 2021–2025), as provider data is consistently released by CMS.

One of our major findings was that access to primary care providers did not increase as much over time as specialty care providers. There is a myriad of potential reasons for this, such as lagging increases in the total number of primary care providers, continued urbanization patterns of providers and populations [47–49], and changes in travel patterns. Lagging increases in the total number of primary care providers from 2016 to 2019 is likely the major contributor to reduced improvements in access in comparison to specialty care, as we found that the total number of primary care-classified providers increased by 11.5% from 2016 to 2019 while the total number of specialty care-classified providers increased by 27.1% from 2016 to 2019. Urbanization of both providers and populations is also a plausible mechanism for the lagging increase in primary care provider access in comparison to specialty care, as Table 1 showed that rural areas had 0% increase in primary care access from 2016 to 2019, while rural specialty care access increased by 35.7%. Our data and analyses suggest that incentives and investment should be focused on both increasing the number of primary care providers overall and especially in rural areas. Between 2016 and 2019, the percentage of the population in contiguous U.S. census tracts utilizing car travel for commuting increased from 90.8% to 91.0%, while commuting via transit decreased from 5.9% to 5.8% and commuting via walking decreased from 3.3% to 3.2%. There are small variabilities by state in these changes. For example, among contiguous U.S. states, Massachusetts census tracts had the largest mean decrease in car commuting (0.41%) from 2016–2019 and also had the second largest mean increase in transit commuting (0.31%) and walk commuting (0.09%). These small changes, even in states with the largest increasing or decreasing deltas by travel modality, are unlikely to be the reason for lagging increases in primary care provider access. We also found that there are no consistent patterns in disparities across ADI quartiles, which is likely due to the fact that roughly 50% of U.S. population living in poverty resides in metropolitan areas [50], where there is stronger spatial access to providers. As spatial access is only capturing the availability and accessibility components of the broader access to care continuum, this does not necessarily indicate that metropolitan populations have affordable, accommodating, or acceptable care.

Improving access to healthcare is a vital component of efforts to reduce the high chronic disease burden and mortality in rural America [34–37,51]. Poor spatial access to care specifically has been linked to higher mortality from emergency surgeries, reduced healthcare utilization, and increases in preventable hospitalizations [4,52–54], but more work is needed to expand analyses into other health outcomes. The data product resulting from our study will provide future research with crucial temporally representative spatial access measures for many different types of providers, which will allow for examination of relationships with a variety of health outcomes. Rural provider shortages due to hospital closures and other factors are worsening over time [55–57], and are likely one of the major contributors to the decreasing spatial access to providers we observed. To strengthen healthcare access in non-metropolitan areas, expansion of the Conrad 30 J-1 visa waiver program may be a viable pathway for states to attract and retain international medical graduates in healthcare shortage areas [58]. The program is currently capped at a 30-waiver limit per state, but a congressional bill introduced in 2023 would increase this limit to 35, which remains lower than the 50 waivers supported by the American Medical Association [59,60]. Domestic medical graduate loan forgiveness programs may also be useful to reduce access disparities in non-metros, yet are often restricted to primary care providers practicing in healthcare shortage areas [61–63]. In our analyses of pulmonology, nephrology, and cancer care, there were zero providers per 100,000 population in small town and rural-coded tracts in 2016 with no increase by 2019. Thus, expanding loan forgiveness programs in shortage areas to include specialty care providers may further improve access to care for non-metropolitan populations [64]. As spatial access was lower both cross-sectionally and over time for non-metropolitan areas, the importance of telehealth in these areas may only be increasing going forward. Though the ‘digital divide’ between urban and rural areas of the U.S. is improving [65,66], access to computers, internet, and smart phones for rural residents remains poorer than in more urbanized areas [67–69], potentially complicating widespread rollout of rural telemedicine capabilities. Other specific barriers include the willingness of healthcare organizations to implement telemedicine in remote areas, the lack of hospital-side digital infrastructure to implement telemedicine, telemedicine reimbursements, provider knowledge of and training for telemedicine, and telemedicine quality assurance [70]. The U.S. Federal Government has recognized barriers and introduced grant programs through agencies to target telemedicine shortcomings, such as the Federal Communication Commission’s Rural Health Care Program and U.S. Department of Agriculture Rural Development’s Distance Learning and Telemedicine Grants program [71,72]. The 2021 congressional infrastructure law also contained provisions for improving rural telemedicine access by narrowing the digital divide [73], but the real-world effects are not yet fully realized. Further local, state, or federal policy steps to tighten the digital divide may be warranted if telemedicine benchmarks are not met. In addition to telemedicine, mobile health clinics are another pathway to both improve access to care and serve as gateways for underserved populations to enter the broader healthcare system [74]. U.S. mobile health clinics are typically located in densely populated areas [74], so improving geographic equity (i.e., expanding clinics in non-metropolitan areas) may serve as one further avenue to improve access to care for rural populations.

Our study had several limitations. We utilized transit network files that are largely maintained by local municipalities with varying levels of completeness and update frequency. This could have resulted in underestimated transit availability, particularly in rural and underfunded areas due to less frequent or incomplete transit network updates. We combine or link a number of separate U.S. Federal Government databases including population, ADI, commuting patterns, and tract/BG boundaries from the U.S. Census Bureau, RUCA codes from USDA, and provider locations from CMS NDFs. Though the government datasets originate from three different agencies, they have geographic interoperability (i.e., all are geographically referenced) and these datasets have been used in conjunction in the literature for health services research [1,2]. Similarly, OSM data and GTFS files (transit files sourced from BusMaps) have been used in health services literature and in conjunction with Census Bureau population data [3]. All of our databases were able to be combined or linked via census tract and block group Federal Information Processing Standard (FIPS) codes. For OSM and GTFS files, utilization of other proprietary mapping or transit databases (e.g., Google Maps Routes API or Transitland API) would have been prohibitively expensive to perform the national routing procedures we undertook in this analysis. An important limitation of the CMS dataset we utilized is that certain providers in the dataset have multiple specialties (i.e., primary and secondary specialties) and in order to be comprehensive, providers with any primary or secondary specialties that fit to a given provider classification category were retained in that category. For example, “internal medicine” is a specialty that is included under the umbrella term “primary care,” but these providers frequently have other primary or secondary specialties. There was no avenue to comprehensively determine in the CMS dataset whether these providers serve primarily as specialists or primarily as primary care providers or serve as specialty care providers but also perform primary care services in their role as internists. This could have resulted in overestimation of the number of primary care providers, but the exact overestimation was not possible to compute because we cannot discern specific provider services available, or care rendered outside of specialties included in the CMS database. Nevertheless, the 2016 count of 388,595 primary care providers included in our analysis generally reflects estimates provided in the literature [75]. This overestimation could also apply to the specialist provider types we studied, as specialist providers with an “internal medicine” primary or secondary specialty may actually primarily serve as primary care providers or also perform primary care services in addition to services classified as specialist. One further limitation of the CMS data utilized is that duplicates of individual providers were required to be included in our analysis, as done in previous work using the CMS provider database [6,7], in order to capture all provider locations. This analytical decision was required because many providers have separate hospital and clinic locations, and the CMS data utilized has no information about how providers split their services between the separate locations in which they practice medicine. Therefore, spatial access to all provider types studied may also be overestimated through this avenue. We also used a maximum catchment area size of 30 minutes based on literature support [2,3,5]. A larger catchment size may be warranted for specialized care, as patients may be willing to travel further. There is a lack of empirical avenues to optimize the maximum catchment size, which could be addressed in future methodological development. We also did not include bicycle travel in computing spatial access to care, as commuting by bicycle is relatively rare in the U.S. [76,77]. Within the vein of population travel preferences, we used a proxy measure, census commuting patterns, to capture how populations may travel to their providers. Commuting patterns may not fully capture how populations actually prefer to travel to providers, but because there are no national tract or BG-level measures of population travel preferences to providers, this was only nationally representative option for composite measure weighting of travel modalities. Despite limitations, our study contributes new knowledge about rural disparities in multi-modal access to primary and specialty care and provides the first temporal spatial access to care dataset for multiple provider types in U.S. neighborhoods.

## Supporting information

S1 File Supplementary methods. (DOCX)

S2 File Data for figures and web map. (XLSX)

S3 File STROBE checklist. (DOCX)

## Abbreviations

P2P: provider-to-population
NDF: National Downloadable File
CMS: Centers for Medicare and Medicaid Services
BG: block group
FCA: floating catchment area
E2SFCA: enhanced two-step floating catchment area
ACS: American Community Survey
RUCA: Rural-Urban Commuting Area
ADI: area deprivation index
