# Correction: Disparities in multi-modal spatial access to primary and specialty care in U.S. neighborhoods: A cross-sectional and temporal analysis of health services

**DOI:** 10.1371/journal.pone.0339502

**Published:** 2025-12-22

**Authors:** R. Blake Buchalter, Paul R. Gunsalus, Madeleine M. Blazel, Michael W. Kenyhercz, Jarrod E. Dalton

There are errors in the author affiliations. The correct affiliations are as follows:

R. Blake Buchalter^1,2,3^, Paul R. Gunsalus^3^, Madeleine M. Blazel^3,4^, Michael W. Kenyhercz^3^, Jarrod E. Dalton^3^

**1** Department of Epidemiology, University of Alabama at Birmingham School of Public Health, Birmingham, Alabama, United States of America, **2** Genomic Medicine Institute, Cleveland Clinic, Cleveland, Ohio, United States of America, **3** Department of Quantitative Health Sciences, Cleveland Clinic, Cleveland, Ohio, United States of America, **4** Cleveland Clinic Lerner College of Medicine, Case Western Reserve University, Cleveland, Ohio, United States.
