# Supplementary material for: Disparities in multi-modal spatial access to primary and specialty care in U.S. neighborhoods: A cross-sectional and temporal analysis of health services
Source: PLoS One. 2025 Sep 17;20(9):e0330427. doi: 10.1371/journal.pone.0330427 (PMC12443297; doi:10.1371/journal.pone.0330427)
Supplement: S1 File — (DOCX) [file pone.0330427.s001.docx]

**Supplementary Methods**

**Geocoding**

Geocoding of physician street addresses was primarily completed via Census Geocoder batch geocoding and OSM Nominatim single address geocoding in the tidygeocoder R package, with ArcGIS World Geocoding Service in ArcGIS Pro version 2.4 serving as a reserve method to geocode unmatched addresses. Spatial points of physician locations were spatially joined to a geodatabase containing population counts then were separately linked to census tract and block group polygons. For physicians with multiple practice locations, such as those with both a permanent practice address and separate clinic addresses, all locations were retained.

**Travel time calculation**

Travel time calculations were computed for population centers of census tracts and block groups within 2 hours of one another. The first bounding box was set to have a center of latitude of 39.50, and longitude of -98.25, which is the geographic center of the contiguous U.S. Next, bounding boxes were calculated using the corners of the original bounding box as centroids and the left and right midpoints of original bounding box as centroids. Bounding boxes were calculated repeatedly with this process until, the entire 48 contiguous US states were all included. Travel times were calculated across 210 overlapping bounding boxes across the contiguous lower 48 states, each of which having a radius of 150 miles. If a location did not have a calculated travel time (within 2 hours) to any other location within bounding box, the location coordinates were “jittered” (randomly perturbed) with a maximum difference of ±0.05 degrees latitude and longitude. If a jittered point was calculated to have a travel time to any other point in the census tract or block group, then the jittered point was used for all future locations. If still no travel times were calculated within 2 hours, we further enhanced the difference to 0.1 degrees latitude and longitude. If still no travel times were calculated, it was assumed that the location was not with 2 hours of any other location. For bounding boxes with more than 2500 possible locations, this process was first limited to a random of 2500 possible locations with the bounding box to identify a non-missing travel time. Jittering was performed because the travel time operation relies on “snapping” of population center points of tracts and block groups to travel networks (i.e., assigning of points to the closest road or transit network), and if points are sufficiently far from travel networks, then no travel times are able to be calculated. Since changes in infrastructure and traffic density impact travel times, we fixed the date and time for car and walking at June 13, 2017, 8:00 AM EDT. The minimum travel time for each ‘from’ and ‘to’ location across the bounding boxes was used as the final travel time.

For transit times, travel time was calculated for each location at each hour, from 7 AM to 7PM standard time within the time zone for bounding box centroid on December 27, 2023. The median travel time across the 13-hour span within each bounding box was used, and then further consolidated to the minimum travel time across the bounding boxes. All transit times faster than walking times by at least 1 minute were used for the transit travel time calculation, otherwise the walking time was assumed the same as transportation time.

The files used for calculating travel times was downloaded using the osmextract R package [1], using the OpenStreetMap “us-latest” dataset from Geofabrik on November 10, 2023 [2]. Osmium version 1.16.0 was used to extract the data within each bounding box. All transportation files (GTFS) for the United States were downloaded from gtfs.pro, now known as BusMaps, on February 28, 2024. All GTFS files used in the calculation had to have a start time prior to December 27, 2023 and an end date after December 27, 2023. Any GTFS file with a bounding box greater than 250,000 square kilometers was not considered in the calculation; only 6 of the 479 files did not satisfy this condition. This bounding box limit was imposed due to the fact that long-range transit modalities, such as cross-country Amtrak lines are exceedingly unlikely to be used for accessing providers. For computation of car, walking, and transit travel times between tracts and block groups, we used the *travel_time_matrix* function in the r5r R package.

**Spatial access computation procedures**

We then utilized the *floating_catchment_area* function in the r5r R package with catchment threshold steps of 10, 20, and 30 minutes and distance decay weights of 1.0 at 0-9 minutes, 0.666 at 10-19 minutes, and 0.333 at 20-29 minutes, and 0 at 30 minutes. There is no generally accepted or empirical way to optimize maximum catchment size in the context of E2SFCA for all types of providers, so we utilized a maximum of 30 minutes for all provider classifications, as frequently done for primary care.

**Mathematical notation for proportional weighted average**

The mathematical notation for our proportional weighting of provider-to-population ratios by travel modality can be represented as follows:

$$\bar{x}=\frac{\sum_{i=1}^{n} x_{i}w_{i}}{\sum_{i=1}^{n} w_{i}}$$

Or more simply as follows:

$$\bar{x}=\frac{\left( x_{1}w_{1} \right)+\left( x_{2}w_{2} \right)+\left( x_{3}w_{3} \right)}{w_{1}+w_{2}+w_{3}}$$

where $\bar{x}$ is the weighted average of census tract or block group P2P ratios, $w$ is a vector of proportional weights applied to car, transit, and walking modalities based on census tract commuting patterns; $x$ is a vector of census tract provider-to-population ratios for car, transit, and walking modalities.

**Supplementary References**

1. Gilardi A, Lovelace R. osmextract: Download and Import Open Street Map Data Extracts [Internet]. 2023. Available from: https://docs.ropensci.org/osmextract/, https://github.com/ropensci/osmextract

2. Bennett J. OpenStreetMap. Packt Publishing Ltd; 2010.

3. Luo W, Wang F. Measures of spatial accessibility to health care in a GIS environment: synthesis and a case study in the Chicago region. Environment and Planning B: Planning and Design. 2003;30(6):865–84.

4. Guagliardo MF. Spatial accessibility of primary care: concepts, methods and challenges. International Journal of Health Geographics. 2004 Feb 26;3(1):3.

5. Luo W, Qi Y. An enhanced two-step floating catchment area (E2SFCA) method for measuring spatial accessibility to primary care physicians. Health & Place. 2009 Dec 1;15(4):1100–7.
